# The Road to Unfreedom: Violence and Multidimensional Poverty Among Young Australian Women

**DOI:** 10.1177/10778012251347607

**Published:** 2025-06-11

**Authors:** Alice Campbell, Ella Kuskoff, Janeen Baxter, Deborah Loxton

**Affiliations:** 1 ARC Centre of Excellence for Children and Families over the Life Course, Institute for Social Science Research, 1974 University of Queensland, Brisbane, QLD, Australia; 2 ARC Centre of Excellence for Children and Families over the Life Course, School of Social Science, 1974 University of Queensland, Brisbane, QLD, Australia; 3 Centre for Women's Health Research, University of Newcastle, Newcastle, NSW, Australia

**Keywords:** coercive control, multidimensional poverty, life course, longitudinal

## Abstract

Our study examined the cumulative impacts of violence from childhood through to adulthood on young women's multidimensional poverty risk. We analyzed six waves of data from the 1989–1995 birth cohort of the Australian Longitudinal Study on Women's Health (*N* = 11,224 women), estimating multinomial logistic regression and fixed-effects logistic regression models. Our measure of multidimensional poverty captured women's deprivations in material, education, employment, health, and social/relationship domains. Women exposed to violence in childhood were more likely to be in multidimensional poverty across young adulthood, and more likely to be revictimized at the hands of intimate partners. Coercive control significantly increased women's multidimensional poverty risk.

Eradicating violence against women is arguably not only a human rights imperative, but also a fundamental step in reducing poverty. There are strong, bidirectional relationships between women's vulnerabilities to poverty and their experiences of violence (Cameron & Tedds, 2021). In one direction, women and children living in poverty are more likely to be the victims of violence and abuse in the home (Herrenkohl et al., 2022; Lacey et al., 2022). In the other direction, the risk of becoming poor is significantly heightened among women who are victims of violence during childhood (Bunting et al., 2018) or adulthood (O’Connor & Nepomnyaschy, 2020). Powerlessness, social isolation, and stress are at the core of both poverty and violence, and together they severely constrain women's agency (Goodman et al., 2009).

In this paper, we extend evidence on the relationships between violence against women and poverty in two important directions. First, we look beyond unidimensional measures of poverty to examine the ultimate cause and consequence of gender-based violence: women's unfreedom. Consistent with feminist understandings of oppression (e.g., Frye, 1983; Young, 2021), we define unfreedom as a state of limited agentic power; that is, an inability to take actions based on one's values, desires, and best interests due to external/social constraints (Campbell, 2009; Sen, 2001; Stark, 2007).^1^ While unfreedom is experienced subjectively, it also has a clear material basis and can therefore be operationalized using the multidimensional poverty framework (Alkire & Foster, 2011). Although numerous scholars to have argued for a multidimensional approach to understanding the impacts of violence on women (Gilroy et al., 2018, 2020; Nussbaum, 2005; Stark, 2007), very few quantitative studies have risen to this challenge (recent exceptions being Sharp-Jeffs et al., 2018; van der Velden et al., 2021). Thus, the first empirical contribution of our study is our use of a multidimensional measure of poverty to more accurately quantify the impacts of violence on women.

In addition, the life course perspective (Elder, 1995) and cumulative disadvantage theory (Dannefer, 2003) motivate us to consider the longitudinal dynamics of gender-based violence and women's unfreedom. Most prior research on this topic has focused on discrete periods of the life course. This compartmentalization has arguably hindered our understanding of the true scope of the problem and the development of effective solutions as a result (Herrenkohl et al., 2022). Exposure to the cultural and material processes of the gender order begins in childhood. For some girls, this will include being the victim of domestic, family, and sexual violence (DFSV), or witnessing the perpetration of such violence against their mothers. The impacts of this violence can reverberate across women's lives (e.g., Cervantes & Sherman, 2021). Thus, the second empirical contribution of our study is our examination of the longitudinal dynamics of women's exposure to violence and multidimensional poverty from childhood through young adulthood.

## Gender-Based Violence and Multidimensional Poverty

Several scholars have made a strong case for taking a multidimensional approach when considering the effects of DFSV on survivors (e.g., Gilroy et al., 2018, 2020; Nussbaum, 2005; Stark, 2007). Such an approach is appropriate for two reasons. First, because perpetrators of coercive control deliberately target multiple domains of women's lives. This can involve constraining a woman's access to economic, psychological, social, and community resources to reduce her “space for action”^2^[Bibr bibr35-10778012251347607] (Stark, 2007). For this reason, Stark (2007) referred to coercive control as a “liberty crime”—in other words, a restriction on a woman's capabilities and violation of her right to freely determine her own life. Second, because of the potentially “interlocking effects of various areas of functioning on one another” (Nussbaum, 2005). A woman experiencing high levels of physical or psychological trauma as the result of violence is less likely to achieve her desired functioning in domains such as employment, education, and political participation. Likewise, a woman who has been denied access to education, employment, income, and wealth by an abusive partner is less likely to be thriving in terms of her physical and mental health.

While there is a strong theoretical argument for using a multidimensional poverty framework to examine the impacts of violence on women, only a small number of empirical studies have taken such an approach. In one recent example, van der Velden et al. (2021) analyzed data from two Dutch population-based surveys. They compared victim-survivors of intimate partner violence (IPV) to a matched sample of nonvictims across twelve negative outcomes, including poor physical and mental health, unemployment, financial and legal problems, and a lack of social and emotional support. Almost half (47%) of victim-survivors reported six or more negative outcomes compared to just 5% of matched nonvictims.

In a similar vein, Sharp-Jeffs et al. (2018) created the Space for Action Scale to capture the impacts of coercive control across seven domains of women's lives: psychological, efficacy, economic, physical, social support and relationships, wider community, and parenting. This scale was administered to a sample of 100 women in the United Kingdom who had accessed domestic violence services and agreed to take part in the longitudinal study. At baseline, a strong cross-sectional association was found between women's total scores on the space for action scale and their scores on a coercive control scale. Specifically, higher coercive control victimization in their current relationship was related to lower space for action. However, once women left their abusive partners their space for action increased and the association with past violence severity was no longer significant. The authors concluded that their space for action scale represents a first step in measuring the extent to which women “are able to restore agency and freedom” after leaving a violent relationship (Sharp-Jeffs et al., 2018, p. 183).

In another example, Ford-Gilboe et al. (2009) analyzed data from a community sample of Canadian women who had left an abusive partner. Using structural equation modelling, they found that the severity of women's past experiences of IPV was negatively related to their current physical and mental health—both directly, and indirectly via deficits in women's combined personal, social, and economic resources. The authors noted that no specific type of resource emerged as a significant mediator on its own; rather, it was the combination of resource deficits that proved important. The authors interpreted this as reflecting “the coherence of women's lives, reminding us that distinguishing among types of resources is largely an analytic exercise given that, in real life, they are experienced as intertwined” (Ford-Gilboe et al., 2009, p. 1027).

Together, these studies provide empirical support for the multidimensional approach to measuring poverty among survivors of violence against women. However, all are limited in their focus on adulthood, with none of them accounting for childhood experiences. This is an important gap to address. For many women, their first experience of DFSV is in childhood. In Australia, it is estimated that more than one in four women were physically or sexually abused or witnessed violence against a parent before the age of 15 (Australian Bureau of Statistics, 2023). As we argue in the next section, cumulative disadvantage theory suggests that these early life experiences can impact women's trajectories of multidimensional poverty and their risk of re-victimization in adulthood. Thus, it is imperative to take a longitudinal, life course approach when considering the dynamics of violence and unfreedom in women's lives.

## Cumulative Disadvantage Theory

Cumulative disadvantage refers to “a systemic tendency for interindividual divergence in a given characteristic or capital (e.g., money, health, or status) with the passage of time” (Dannefer, 2003: p. S327). Central to this concept is the tenet that experiences early in the life course have important ramifications on adult outcomes. Intersecting social structures put individuals in positions of relative advantage or disadvantage early in life and shape their trajectories going forward. What can start as a small difference can widen over time as risks and disadvantage accumulate. Cumulative disadvantage is a salient theory for understanding the dynamics of violence and unfreedom in women's lives. DFSV are the product of intersecting structural inequities—most notably gender, but also social class, race and ethnicity, disability, and sexual orientation (Crenshaw, 1991; Tolmie et al., 2024). Early-life experiences of violence can then lead to the accumulation of further risks of violence and disadvantage across women's lives.

There are several pathways through which child abuse or maltreatment can negatively impact adult outcomes. Maltreatment can trigger what has been described as a “cascade of maladaptation” in a child's neurobiological, socioemotional, and cognitive development (Cicchetti, 2013). Mental and physical illness, negative self-concept, emotional dysregulation, difficulty concentrating, and impairments to memory and cognitive processing are some of the documented sequalae of traumatic experiences early in life (e.g., Heim et al., 2010; Op den Kelder et al., 2018). These have clear implications for a person's ability to engage with and complete their education and participate in the labor market. Sexual abuse and violence in the home are also risk factors for youth homelessness and early motherhood, both of which can impede women's socioeconomic attainment (e.g., Chamberlain & Johnson, 2013; McNamara, 2015). Similarly, children who experience maltreatment are at higher risk of being placed into out of home care through the statutory child protection system compared to children without such experiences (Perlman & Fantuzzo, 2013). Being placed in out of home care is in turn strongly linked to adverse life outcomes across multiple domains (Brännström et al., 2017; Kääriälä & Hiilamo, 2017; Sariaslan et al., 2022).

Yet another crucial pathway linking childhood victimization to multidimensional poverty among women is revictimization in the form of IPV. There is growing evidence that being the victim of violence at one stage of the life course increases the risk of being the victim of violence at another stage (Brownell, 2024; Butler et al., 2020; Giraldo-Rodríguez et al., 2022; Herrenkohl et al., 2022; Pezzoli et al., 2024). A recent analysis of data from a large, nationally representative sample from the United Kingdom found that individuals who had experienced one type of abuse as a child were three times more likely to experience intimate partner or sexual violence in adulthood compared to individuals who had not been abused as a child (Butler et al., 2020). For those who had experienced multiple types of child abuse, the comparable odds were six to seven times as high. In a qualitative investigation of women's experiences of the cycle of violence, Cervantes and Sherman (2021) describe “a discursive process of normalization,” with early life experiences of violence (including direct victimization and witnessing their mothers being victimized) socializing women to believe that IPV is normal and expected. Experiences of violence and abuse can also damage a woman's self-esteem, self-identity, and self-confidence, making her more vulnerable to revictimization (Cervantes & Sherman, 2021; Childress, 2013).

Qualitative evidence suggests that the accumulation of violence victimization is often accompanied by the accumulation of disadvantages across other life domains. For example, Farber and Miller-Cribbs (2014) conducted life history interviews with 32 poor, white single mothers living in rural South Carolina in the United States. Growing up, most of these women had witnessed their mothers being physically victimized and/or coercively controlled by their male partners. Many of the women had also survived childhood physical and/or sexual abuse. They described how these early life experiences led to life course trajectories marked by the accumulation of vulnerabilities and “ever greater difficulty in achieving financial, social, and physical security” (Farber & Miller-Cribbs, 2014, p. 527). School disengagement and drop out, leaving home at a young age, early sexual debut, teen pregnancy, and IPV in adult relationships were salient experiences for many. The authors noted that these “stacked vulnerabilities” functioned to “reduce the women's abilities to develop human and social capital and accumulate assets” (Farber & Miller-Cribbs, 2014, p. 532). We expect the results of our analyses to reveal a similar process: early-life exposure to violence leading to an increased risk of multidimensional poverty in young adulthood, punctuated by a higher risk of (more severe) IPV that compounds pre-existing disadvantage. To our knowledge, our study will be the first to test this quantitatively using longitudinal data from a large, national dataset, providing robust and generalizable evidence of what others have theorized and demonstrated qualitatively.

## Method

### Data and Sample

To achieve our study aim, we analyzed six waves of data from the 1989–1995 birth cohort of the Australian Longitudinal Study on Women's Health (ALSWH). In 2012–2013, 17,011 women born in the years 1989–1995 were recruited into the study via promotions in traditional and online/social media (e.g., Facebook),^3^ in person, snowballing, and peer referral. To be eligible to participate, women needed to be born in the target years and eligible for Medicare, which is the Australian health insurance scheme covering all citizens and permanent residents (i.e., approximately 96% of the Australian population). The first wave of data was collected from the women in 2013 (Wave 1), with subsequent waves of data collection in 2014 (Wave 2), 2015 (Wave 3), 2016 (Wave 4), 2017 (Wave 5), and 2019 (Wave 6). All data were collected via online surveys.

In Wave 1, the women were aged 18–24 years (*M* = 20.6, *SD* = 1.7). The sample was found to be broadly representative of the population of Australian women born at the same time, with the exception that tertiary-educated women were somewhat overrepresented and women from a non-English speaking background were underrepresented (Loxton et al., 2018). In our final analytic sample, 44.7% of person-year observations came from women with a university qualification, and 73.9% came from women living in a metropolitan area. Meanwhile, 91.9% of women in our sample were born in Australia, 5.0% were born in another English-speaking country, and 3.1% were born in a non-English-speaking country.

### Measures

#### Violence Victimization in Childhood

Exposure to DFSV during childhood was measured using 16 binary-response items adapted from the Adverse Childhood Experiences (ACEs) study (Felitti et al., 1998). These questions were asked in Wave 3 of the survey, and again in Waves 5 and 6 for those missing from Wave 3. We used responses to these items to create four time-invariant binary variables for childhood exposure to (1) sexual abuse, (2) physical abuse, (3) psychological abuse, and (4) witnessing violence against a parent. We then created an ordinal variable capturing the number of different types of violence experienced in childhood, with four categories: (1) no violence, (2) one type of violence, (3) two types of violence, and (4) three or four types of violence.

#### IPV in Adulthood

We used responses to questions about IPV from each of the six study waves. Women were asked if they had experienced 11 different types of violent behavior at the hands of a current or former partner in the previous 12 months. These questions were taken from an abbreviated form of the Community Composite Abuse Scale (CCAS: Loxton et al., 2013), which was derived from the Composite Abuse Scale (CAS: Hegarty et al., 1999). Although coercive control was not widely recognized when the CAS was developed, the CAS does include measures of common coercive and controlling behaviors. These include sexual violence, verbal abuse and intimidation, harassment, stalking, social isolation, and economic abuse. In total, seven of the 11 abusive behaviors measured in the survey were non-physical, one was sexual assault, and the other three were forms of physical assault (e.g., being hit, kicked, thrown, beat up, or attacked with a weapon).

Using these items, we created a time-varying ordinal variable capturing the presence and severity of IPV in the previous 12 months. We considered IPV to be more severe if the probability of coercive control was higher. In deciding on cut-offs for the levels of our ordinal variable, we drew on the broad approach taken by Johnson et al. (2014) who created a measure of coercive control using secondary data from the U.S. National Violence Against Women Survey. We created the following categories based on responses to the 11 items: (0) No IPV (0 items reported), (1) IPV with no/low coercive control (one to two items reported), (2) IPV with moderate coercive control (three to five items reported), and (3) IPV with high coercive control (six+ items reported).

In addition, we created a time-invariant variable capturing the most severe IPV reported across all study waves for each woman. This time-invariant variable had the following mutually exclusive categories: (0) never reported any IPV, (1) only ever reported IPV with no/low coercive control, (2) reported moderate coercive control at least once but never high coercive control, and (3) reported high coercive control at least once.

#### Multidimensional Poverty

To identify the indicators and domains for our multidimensional poverty index, we followed the broad approach taken in previous Australian studies (Martinez & Perales, 2017; Scutella et al., 2013). Unfortunately, the ALSWH surveys did not contain repeated measures relevant to the “community” domain, which includes constructs such as neighborhood quality and civic participation. Further, we did not include the “safety” domain in our measure of multidimensional poverty given that violence victimization was to be our predictor (i.e., we did not want to have violence victimization on both sides of the equation), and the dataset did not contain any other measures of safety such as perceived safety or fear. This left us with five domains: material, employment, education, health, and social/relationships.

For each of these five domains, we were able to identify between one and three variables that were measured in every wave. These were: ability to manage on current income, stress about money, and possession of a government low-income healthcare card (material domain); unemployment status and usual number of hours worked (employment domain), highest qualification attained (education domain), general self-rated health and psychological distress (health domain), and stress about relationships with parents, other family members, and friends (social/relationships domain: see Appendix Table A1 for more details).

We used the Alkire-Foster method^4^ to create our multidimensional poverty index (Alkire & Foster, 2011; see also Alkire & Santos, 2013). This is a counting approach and dual cutoff method of identification. First, deprivation cut-offs are assigned for each indicator, as outlined in Appendix Table A1. Second, the overall poverty cut-off is set. We assigned equal weight to indicators within each domain. The five domains composing the index were also weighted equally. Index scores ranged from 0 to 1 (*M* = .13, *SD* = .15). In keeping with prior Australian studies, we decided that a person would be considered multidimensionally poor if they were deprived on the equivalent of at least two domains (index score of .4, approximately two standard deviations above the mean). This resulted in a multidimensional poverty prevalence of 7.8% (*n* = 4,152) across observations in our sample. Almost one in five women (19.6%) were in multidimensional poverty at least once across the six study waves. The largest contribution to the index came from the material domain (28.8%), followed by employment (23.6%), education (20.1%), health (16.9%), and social/relationship (10.6%) domains. Table 1 displays descriptive statistics for the index, indicators, and domains for the full sample and according to multidimensional poverty status.

**Table 1.** Descriptive Statistics for Domains and Indicators Used to Construct Multidimensional Poverty Index.

	All		In MDP		Not in MDP	
	*M (SD)*	*%* Obs	*M (SD)*	*%* Obs	*M (SD)*	*%* Obs
Multidimensional poverty index	.13 (.15)		.51 (.11)		.10 (.11)	
Material domain	.25 (.31)		.73 (.28)		.22 (.28)	
Always difficult/impossible to manage on income		18.4%		68.2%		14.2%
Very/extremely stressed about money		33.5%		82.4%		29.4%
Has a low-income healthcare card		25.5%		68.6%		21.8%
Employment domain	.13 (.28)		.60 (.41)		.09 (.23)	
Has been unemployed for more than 6 months		8.2%		48.9%		4.7%
Works zero hours in a usual week		16.9%		70.7%		12.4%
Education domain	.11 (.32)		.51 (.50)		.08 (.27)	
Highest education qualification is Year 12 or less		11.2%		50.9%		7.8%
Health domain	.10 (.22)		.43 (.33)		.07 (.18)	
Poor self-rated health		2.8%		18.8%		1.4%
Very high psychological distress		16.7%		66.8%		12.5%
Social/relationship domain	.07 (.17)		.27 (.31)		.05 (.14)	
Very/extremely stressed about relationship with parents		9.7%		32.9%		7.7%
Very/extremely stressed about relationship with other family		5.0%		22.0%		3.6%
Very/extremely stressed about relationship with friends		6.0%		25.6%		4.3%
*N* Observations	53, 127		4,152		48,975	

*Notes*. Australian Longitudinal Study on Women's Health. Women born 1989–1995. Data from Waves 1–6 (2013, 2014, 2015, 2016, 2017, 2019).

Scores for total index and for each domain range from 0 to 1. Indicators are binary. MDP = multidimensional poverty.

While equal weighting of indicators and domains is the most common approach when constructing multidimensional indices, some scholars have argued that it is too arbitrary. We therefore tested the robustness of our results to alternative, data-driven weighting schemes. In the first, domains were weighted according to their factor loadings. In the second, indicators were weighted according to factor loadings, which were estimated from a tetrachoric correlation matrix given the binary nature of the indicators. Table A2 in Appendix 2 shows the factor loadings and assigned weights for each domain and indicator in these alternative specifications. The prevalence of multidimensional poverty was 7.8% under our original specification, compared to 7.5% for Alternative Index 1 and 8.7% for Alternative Index 2. Estimates from fixed-effects logistic regression models of multidimensional poverty as a function of past-year IPV did not differ significantly between the original index and the two alternative specifications, suggesting that our results are robust to alternative weighting schemes (see Appendix 2, Table A2.1).

In addition to the time-varying dummy indicator of multidimensional poverty, we created a time-invariant variable capturing the frequency of multidimensional poverty for each woman across all study waves. We created this variable for each woman by taking the number of waves she was in MDP and dividing it by the number of waves that she participated in the study. This time-invariant variable had the following categories: (0) never in MDP (1) rarely in MDP (up to 25% of study waves), (2) sometimes in MDP (>25% and < = 50% of study waves), and (3) often in MDP (more than 50% of study waves).

#### Covariates

We controlled for financial hardship growing up (family's ability to manage on income when in high school: easy/not too bad/difficult some of the time/difficult all the time/impossible/don’t know) country of birth (Australia/other English-speaking country/non-English-speaking country)^5^ and current location of residence (major city/inner regional/outer regional or remote/overseas from Australia) in our statistical models. These controls were selected due to their possible confounding effects on the relationship between violence and multidimensional poverty.^6^

### Statistical Analyses

All analyses were conducted using Stata 18. To estimate associations between time-invariant predictors (e.g., childhood DFSV) and outcomes (e.g., frequency of MDP), we used multinomial logistic regression models. When our predictor of interest was time-varying (past-year IPV), we estimated a fixed-effects logistic panel regression model to provide more robust inferences of causality. We provide equations and describe our modelling approaches in more detail in the Results section.

The initial dataset comprised 63,163 observations from 17,010 women, and our final analytic sample comprised 53,127 observations from 11,224 women (median of five observations per woman). The most common source of missing data was the childhood violence variable, which was only asked in Waves 3, 5, and 6 of the study. In total, 5,471 women (32% of the Wave 1 sample) were not present in any of these waves—in most cases, because they exited the study after Waves 1 or 2. This level of attrition is consistent with that observed in many other large cohort studies (see Watson & Wooden, 2009). While nonrandom attrition can bias estimates of population prevalences, there is evidence that it does not bias estimates of associations between variables (e.g., Gustavson et al., 2012; Saiepour et al., 2019). This is reassuring given that our study is focused on the latter rather than the former.

To investigate the potential impact of attrition on our results, we conducted a series of analyses using data from all women who participated in Wave 1. First, we estimated a logistic regression model with a binary indicator for attrition (defined as exiting the study after Wave 1 or Wave 2) as the outcome. We found that the odds of attrition were significantly higher for women in multidimensional poverty in Wave 1 compared to women who were not in multidimensional poverty in Wave 1 (*OR* = 1.72, *p* < .001). Based on our model, the predicted probability (average marginal effects) of attrition for a woman in multidimensional poverty in Wave 1 was 38.9%, compared to 27.1% for a woman who was not in multidimensional poverty.

We also found that past-year IPV was associated with increased odds of attrition. The predicted probability of attrition for a woman who had not experienced IPV in the previous 12 months was 27.8%. In contrast, predicted probabilities of attrition were significantly higher for women who had experienced IPV with no/low coercive control (31.9%), moderate coercive control (35.7%), and high coercive control (33.8%) in the past year. These findings are consistent with prior evidence that indicators of disadvantage and vulnerability, such as low education, unemployment, and poor health, increase the likelihood of attrition from longitudinal studies such as the ALSWH (Campbell et al., 2020; Rothenbühler & Voorpostel, 2016). Given these findings, prevalence estimates for both IPV and multidimensional poverty arising from our study are likely to be biased downwards—in other words, the true population prevalences are likely to be higher than we report here.

To investigate the potential impacts of attrition on our estimates of associations between violence and multidimensional poverty—the core focus of our study—we compared coefficients from logistic regression models of multidimensional poverty in Wave 1. The first model was estimated on the full sample (i.e., including subsequent attritors), and the second model excluded attritors. Wald tests found no significant differences in the coefficients for IPV estimated for the full sample and the subsample without attritors. For example, the odds ratio of being in multidimensional poverty for women reporting high coercive control compared to no IPV was 5.12 (*SE* = .62) for the full sample and 4.71 (*SE* = .75) for the subsample without the attritors, yielding a *Z* statistic of .42 (*p* = .67). This provides reassurance that attrition is unlikely to bias estimates of associations between variables in our study, which is consistent with prior literature (Gustavson et al., 2012; Saiepour et al., 2019).

## Results

Approximately two-thirds (65%) of women in our sample did not report any domestic, family, or sexual violence growing up, while 18% reported one form of childhood violence, 9.7% reported two forms, and 7.5% reported experiencing three or four forms of violence growing up. Table 2 displays descriptive statistics for the full analytic sample and for each childhood violence group separately. Table 2 shows that women across the childhood violence groups were similar in terms of age and country of birth. Women who grew up exposed to three to four forms of DFSV were slightly less likely than all other groups to live in a major city, and slightly more likely to live in a regional, rural or remote area. Where the groups differed most markedly, however, was in the financial situation of their families when they were growing up. The more forms of violence that a woman was exposed to growing up, the more likely she was to report that her family's ability to manage financially was “difficult all the time” or “impossible” when she was in high school.

**Table 2.** Descriptive Statistics for Full Analytic Sample and by Childhood Exposure to DFSV.

		Childhood Exposure to DFSV			
	All	None	1 Form	2 Forms	3–4 Forms
Country of birth	*%*	*%*	*%*	*%*	*%*
Australia	91.85	92.00	91.81	91.63	90.90
Other English-speaking country	5.03	5.13	4.76	4.93	4.86
Non-English-speaking country	3.12	2.87	3.42	3.44	4.25
Location of residence in Wave 1					
Major city	74.45	74.74	74.88	74.42	70.90
Inner regional	17.01	16.69	16.80	18.10	18.88
Outer regional/remote	7.46	7.32	7.68	6.65	9.14
Overseas	1.09	1.25	.64	.83	1.07
Family's ability to manage on income during high school					
Easy	26.99	33.14	18.97	13.78	6.09
Not too bad	33.94	36.64	32.28	27.20	21.38
Difficult some of the time	27.37	23.88	33.93	34.02	35.36
Difficult all the time	9.51	5.05	13.37	20.85	27.14
Impossible	1.10	.29	.72	2.80	7.40
I don’t know	1.10	1.00	.72	1.34	2.63
Worst IPV reported					
None	64.97	71.53	58.97	51.25	40.26
IPV with no/low coercive control	20.13	18.02	23.04	25.30	24.70
Moderate coercive control	10.08	7.43	12.98	15.51	19.00
High coercive control	4.83	3.02	5.00	7.94	16.03
MDP frequency					
Never	80.38	87.27	75.37	65.93	51.43
Rarely	7.93	6.07	9.81	12.47	13.66
Sometimes	6.79	4.37	8.67	11.54	17.10
Often	4.90	2.29	6.14	10.06	17.81
Age in Wave 1: *M (SD)*	21.2 (1.7)	21.2 (1.7)	21.1 (1.7)	21.2 (1.8)	21.1 (1.7)
*N* Individuals	11,224	7,281	2,018	1,083	842

*Notes*. Australian Longitudinal Study on Women's Health. Women born 1989–1995. Data from Waves 1–6 (2013, 2014, 2015, 2016, 2017, 2019). DFSV = domestic, family, and sexual violence. IPV = intimate partner violence. MDP = multidimensional poverty.

### Childhood Victimization and IPV Severity

A chi-square test of independence revealed a positive, bivariate association between exposure to DFSV in childhood and the severity of IPV experienced in young adulthood (*X*^2^ (9, *N* = 11,224) = 673.5, *p* < .001). Approximately 72% of women from the “no childhood violence” group did not report any IPV across the six study waves, compared to 40% of women exposed to three to four forms of violence growing up. Only 3% of women who grew up free from domestic, family, and sexual violence experienced the most severe form of IPV, characterized by high levels of coercive control, at least once across the six study waves. In contrast, IPV with high coercive control was reported by 8% of women exposed to two forms of violence growing up, and 16% of women exposed to three to four forms of violence growing up.

We estimated a multinomial logistic regression model to test the relationship between childhood exposure to DFSV and the severity of IPV experienced in young adulthood, controlling for childhood poverty, age, country of birth, and geographic location. This model estimates the log odds of each outcome (IPV with no/low coercive control, moderate coercive control, high coercive control) relative to the baseline reference (no IPV). The equation for this model is as follows:

(1) $$\ln\;\left(\frac{\mathrm{Pr}(\mathrm{IPV}\;=j)}{\mathrm{Pr}(\mathrm{IPV}\;=0)}\right)=\beta_{0j}+\overset{3}{\underset{h=1}{\sum }}\;\gamma_{hj}{\mathrm{DFSV}}_{h}+\beta_{j}X\;j=\;1,2,3$$

Here, IPV is the outcome variable (IPV severity), which can take the value *j* or the value 0 (reference category). $\beta_{0j}$ is the intercept specific to outcome *j*; ${\mathrm{DFSV}}_{h}$ represents a set of dummy variables for childhood exposure to DFSV, where *h* = 1 (one type), *h* = 2 (two types), *h* = 3 (three to four types), with “no DFSV exposure” as the reference category; $\gamma_{hj}$ is the set of coefficients for the DFSV dummy variables, specific to outcome *j*; ***X*** is a vector of covariates (childhood poverty, age at Wave 1, country of birth, and location of residence at Wave 1), and $\beta_{j}$ is a vector of coefficients for those covariates, specific to outcome *j*.

Table 3 displays the results of this model and shows a significant, positive relationship between the severity of DFSV experienced as a child and the relative risk of experiencing more severe IPV in young adulthood. For example, the relative risk of experiencing high coercive control compared to no IPV was approximately eight times higher for women exposed to the most severe DFSV growing up compared to women who grew up free from violence. For women who grew up free from violence, the predicted probability (average marginal effects) of experiencing no IPV was 71%, while the predicted probability of experiencing high coercive control at least once was 3%. In contrast, for women who grew up exposed to the most severe DFSV, the predicted probability of experiencing no IPV was 43% while the predicted probability of experiencing high coercive control at least once was 15%.

**Table 3.** Relative Risk Ratios From Multinomial Logistic Regression of Intimate Partner Violence Severity Regressed on Childhood Violence.

	Most Severe IPV Reported Across All Study Waves (Reference = None Reported)					
	IPV with No/Low Coercive Control		Moderate Coercive Control		High Coercive Control	
	RRR	95%CI	RRR	95%CI	RRR	95%CI
Childhood exposure to DFSV						
None (reference)						
1 form	1.515***	1.34, 1.72	2.071***	1.76, 2.44	1.883***	1.47, 2.41
2 forms	1.864***	1.59, 2.19	2.779***	2.28, 3.39	3.291***	2.51, 4.32
3–4 forms	2.250***	1.86, 2.72	4.123***	3.31, 5.13	8.001***	6.20, 10.33
Country of birth						
Australia (reference)						
Other English-speaking country	1.193	.95, 1.51	1.005	.72, 1.39	1.356	.89, 2.07
Non-English-speaking country	.603**	.43, .85	.587*	.37, .92	.584	.30, 1.12
Location of residence in Wave 1						
Major city (reference)						
Inner regional	.956	.84, 1.09	1.132	.96, 1.34	1.329*	1.07, 1.66
Outer regional/remote	1.071	.89, 1.29	1.586***	1.28, 1.97	1.110	.79, 1.56
Overseas	.954	.60, 1.51	.956	.50, 1.82	.174	.02, 1.27
Wave 1 age in years	.976	.95, 1.00	.941**	.91, .98	.926**	.88, .97
Family's ability to manage on income during high school						
Easy (reference)						
Not too bad	.911	.79, 1.05	.949	.78, 1.16	.967	.71, 1.32
Difficult some of the time	1.193*	1.03, 1.38	1.191	.97, 1.46	1.526**	1.13, 2.06
Difficult all the time	1.233*	1.01, 1.51	1.239	.95, 1.62	1.553*	1.07, 2.25
Impossible	2.461**	1.43, 4.23	3.719***	2.09, 6.63	3.266**	1.57, 6.80
I don’t know	1.546	.93, 2.56	1.273	.63, 2.58	3.763***	1.90, 7.44

*Notes*. Australian Longitudinal Study on Women's Health. Women born 1989–1995. Data from Waves 1–6 (2013, 2014, 2015, 2016, 2017, 2019). *N* = 11,224 women. DFSV = domestic, family, and sexual violence. IPV = intimate partner violence. Statistical significance: *p < .05, **p < .01, ***p < .001

As Table 3 shows, the relative risks of experiencing moderate or high coercive control compared to no IPV decreased significantly with age. Consistent with previous research findings (McLachlan, 2023; Strand & Storey, 2018), the relative risks of experiencing moderate or high coercive control were also higher among women living in regional or remote areas compared to those living in a major city. There was also a strong relationship between childhood poverty and the severity of IPV experienced in young adulthood. The relative risks of experiencing all severities of IPV compared to no IPV were significantly higher among women who said that it was “impossible” for their family to manage on their income when they were in high school compared to women who said that it was “easy.”

### Childhood Victimization and Multidimensional Poverty Frequency

A chi-square test of independence revealed a positive, bivariate association between exposure to more severe DFSV in childhood and frequency of multidimensional poverty in young adulthood (*X*^2^ (9, *N* = 11,224) = 963.2, *p* < .001). Approximately 87% of women who grew up free from violence were never observed to be in multidimensional poverty across the six study waves. The comparable figures were 75% for women who experienced one form of DFSV in childhood, 66% of women who experienced two forms, and 51% of women exposed to the most severe childhood DFSV. In contrast, around 2% of women who were not exposed to violence growing up were often in multidimensional poverty in young adulthood, compared to 6% of women exposed to one form of DFSV in childhood, 10% of women exposed to two forms, and 18% of women exposed to three to four forms.

We estimated a multinomial logistic regression model to test the relationship between childhood exposure to DFSV and the frequency of multidimensional poverty experienced in young adulthood, controlling for childhood poverty, age, country of birth, geographic location, and severity of IPV in young adulthood. This model estimates the log odds of each outcome (rarely in MDP, sometimes in MDP, often in MDP) relative to the reference (never in MDP). The equation for this model takes the same form as equation (1) and is as follows:

(2) $$\ln\;\left(\frac{\mathrm{Pr}(\mathrm{MDP}\;=k)}{\mathrm{Pr}(\mathrm{MDP}\;=0)}\right)=\beta_{0k}+\overset{3}{\underset{h=1}{\sum }}\;\gamma_{hk}{\mathrm{DFSV}}_{h}+\beta_{k}X\;k=\;1,2,3$$

Here, MDP is the outcome variable (MDP frequency), which can take the value *k* or the value 0 (baseline reference). $\beta_{0k}$ is the intercept specific to outcome *k*; ${\mathrm{DFSV}}_{h}$ represents a set of dummy variables for childhood exposure to DFSV, with *h* = 1 (one type of DFSV), *h* = 2 (two types of DFSV), *h* = 3 (three to four types of DFSV), and “no DFSV exposure” as the reference category; $\gamma_{hk}$ is the corresponding set of coefficients for the DFSV dummy variables, specific to outcome *k*; ***X*** is a vector of covariates (childhood poverty, age at Wave 1, country of birth, geographic location at Wave 1, and most severe IPV reported), and $\beta_{k}$ is a vector of coefficients for those covariates, specific to outcome *k*.

Table 4 displays the results of this model and shows a significant, positive relationship between the severity of DFSV experienced as a child and the relative risk of experiencing more frequent MDP in young adulthood, all else held constant. For example, the relative risk of being in MDP often compared to never was approximately seven times higher for women exposed to the most severe DFSV growing up compared to women who grew up free from violence. For women who grew up free from violence, the predicted probability (average marginal effects) of never being in MDP was 86%, while the predicted probability of being in MDP often was 3%. In contrast, for women who grew up exposed to the most severe DFSV, the predicted probability of never being in MDP was 62% while the predicted probability of being in MDP often was 12%.

**Table 4.** Relative Risk Ratios From Multinomial Logistic Regression of Multidimensional Poverty Frequency as a Function of Childhood DFSV and Intimate Partner Violence Severity.

	Multidimensional Poverty Frequency (Reference = Never)					
	Rarely		Sometimes		Often	
	RRR	95%CI	RRR	95%CI	RRR	95%CI
Childhood exposure to DFSV (ref = none)						
1 form	1.693***	1.41, 2.03	1.824***	1.49, 2.23	2.384***	1.86, 3.05
2 forms	2.302***	1.85, 2.86	2.489***	1.98, 3.14	3.876***	2.97, 5.05
3–4 forms	3.002***	2.35, 3.83	3.855***	3.04, 4.89	6.936***	5.33, 9.02
Worst IPV reported across survey waves (ref = none)						
IPV with no/low coercive control	1.575***	1.32, 1.87	1.298**	1.06, 1.58	1.750***	1.39, 2.20
Moderate coercive control	2.319***	1.89, 2.85	2.185***	1.74, 2.74	2.954***	2.29, 3.81
High coercive control	2.717***	2.04, 3.62	4.194***	3.22, 5.46	5.414***	4.04, 7.26
Country of birth (ref = Australia)						
Other English-speaking country	.957	.67, 1.36	1.163	.78, 1.74	1.449	.95, 2.22
Non-English-speaking country	.666	.40, 1.10	1.022	.62, 1.69	.587	.28, 1.23
Location of residence in Wave 1 (ref = major city)						
Inner regional	1.458***	1.22, 1.74	1.513***	1.25, 1.83	1.583***	1.27, 1.98
Outer regional/remote	1.224	.94, 1.59	1.419*	1.08, 1.86	1.677***	1.24, 2.27
Overseas	1.184	.60, 2.32	.810	.32, 2.04	.666	.20, 2.20
Wave 1 age in years	.873***	.84, .91	.839***	.80, .88	.874***	.83, .92
Family's ability to manage on income during high school						
(ref = easy)						
Not too bad	1.199	.97, 1.49	1.503**	1.13, 2.01	1.909***	1.30, 2.79
Difficult some of the time	1.402**	1.13, 1.75	2.222***	1.68, 2.94	2.847***	1.97, 4.12
Difficult all the time	1.604**	1.20, 2.14	3.810***	2.76, 5.25	4.872***	3.26, 7.28
Impossible	1.738	.90, 3.37	3.616***	1.89, 6.90	5.629***	2.90, 10.94
I don’t know	2.369**	1.25, 4.49	2.886**	1.30, 6.40	8.974***	4.53, 17.79

*Notes*. Australian Longitudinal Study on Women's Health. Women born 1989–1995. Data from Waves 1–6 (2013, 2014, 2015, 2016, 2017, 2019). *N* = 11,224 women. DFSV = Domestic, family, and sexual violence. IPV = Intimate partner violence. Statistical significance: *p < .05, **p < .01, ***p < .001

As Table 4 shows, many of the model covariates were also significant predictors of MDP frequency. The relative risks of being in MDP sometimes or often compared to never were significantly higher among women who experienced more severe IPV, lived in a regional or remote area, were younger, and who experienced poverty in their family growing up.

### The Effects of IPV on Multidimensional Poverty

The results displayed in Table 4 suggest a significant and positive relationship between exposure to more severe IPV and the frequency of MDP in young adulthood. However, these estimates are potentially biased by time-invariant unobserved confounding variables. In other words, they could be biased by a failure to account for stable, individual characteristics associated with both the independent variable (IPV severity) and the dependent variable (MDP frequency). One common approach to overcoming this when analyzing longitudinal (panel) data is the use of fixed-effects models, which focus on within-individual variation. In our case, this involves modeling how within-woman changes in exposure to IPV are associated with changes in multidimensional poverty status.

Fixed-effects models are generally considered a more robust test of causality as they account for unobserved, time-invariant heterogeneity. However, this comes at a cost and relies on several assumptions (Wooldridge, 2010). Drawbacks of the fixed-effects model include the inability to estimate coefficients for time-invariant variables and the discarding of information on between-individual differences (leading to reduced efficiency).^7^ Further, the fixed-effects model relies on the assumption that the unobserved heterogeneity is constant within individuals over time—something that is difficult to test.

Considering both the advantages and drawbacks of the fixed-effects approach, we use it here to augment the results of our prior analyses and provide a more stringent—if still imperfect—test of a causal relationship between IPV and multidimensional poverty. The equation for this model is as follows:

(3) $$\ln\left(\frac{Pr(M_{it}=1)}{\mathrm{Pr}(M_{it}=0)}\right)=\alpha_{i}\;+\overset{3}{\underset{\;j=1}{\sum }}\;\gamma_{j}{\mathrm{IPV}}_{\;jit}\;+\beta X_{it}$$

Here, $\left(Pr(M_{it}=1)\right)$ refers to the probability of being in multidimensional poverty for individual *i* at time *t*; $\alpha_{i}$ is the individual fixed effect capturing unobserved, time-invariant characteristics; ${\mathrm{IPV}}_{\;jit}$ represents a set of dummy variables indicating past-year exposure to IPV at level *j*, where *j* = 1 (no/low CC), *j* = 2 (moderate CC), *j* = 3 (high CC), with “no IPV” as the reference category; $\gamma_{j}$ is the corresponding set of coefficients for the IPV dummy variables; $X_{it}$ is a vector of time-varying covariates (age, age-squared, and location of residence); and β is a vector of coefficients associated with these covariates.

Results of the fixed-effects logistic regression model are displayed in Table 5 and show that the odds of being in multidimensional poverty were approximately 1.7 times higher for a woman if she had experienced moderate coercive control in the past year, and 1.6 times higher if she had experienced high coercive control in the past year, compared to when she had experienced no IPV in the past year. This provides more robust evidence that gender-based violence leads to multidimensional poverty in some women.

**Table 5.** Results of Fixed-Effects Logistic Regression Model of Multidimensional Poverty as a Function of Past-Year Intimate Partner Violence.

	OR	95%CI
Past-year intimate partner violence		
None (reference)		
IPV with no/low coercive control	1.103	.94, 1.29
Moderate coercive control	1.698***	1.38, 2.08
High coercive control	1.581**	1.19, 2.11
Age	.500***	.37, .68
Age-squared	1.011**	1.00, 1.02
Location of residence		
Major city (reference)		
Inner regional area	1.338**	1.08, 1.66
Rural/remote area	1.003	.72, 1.39
Overseas	.876	.50, 1.53
*N* observations	9,417	
*N* Individuals	1,981	

*Notes*. Australian Longitudinal Study on Women's Health. Women born 1989–1995. Data from Waves 1–6 (2013, 2014, 2015, 2016, 2017, 2019). IPV = intimate partner violence. Statistical significance: **p < .01, ***p < .001.

### Additional Analyses

In additional analyses, we re-estimated some of our models using a time-invariant measure of IPV frequency instead of IPV severity (full results are available from the authors upon request). A multinomial logistic regression of IPV frequency as a function of childhood DFSV showed a very similar pattern of results to the model of IPV severity displayed in Table 3. There was a significant, positive relationship between the severity of DFSV experienced as a child and the risk of experiencing more frequent IPV in young adulthood. Likewise, a multinomial logistic regression model of MDP frequency as a function of IPV frequency showed very similar results to those found for IPV severity reported in Table 4. The strong overlap between the two sets of results is not surprising given the large and positive correlation between IPV severity and IPV frequency (*r* = .86, *p* < .001).

A second set of additional analyses explored the impacts of childhood DFSV and IPV severity on the individual indicators of multidimensional poverty that were used to construct our index. The results of these analyses are displayed in Appendix 3, Table A3. Controlling for childhood poverty and other covariates, women who experienced any DFSV growing up were more likely to be deprived on each and every indicator in Wave 1 compared to women who grew up free from violence; and the more severe the violence in childhood, the larger the odds of being deprived. The strongest associations were observed in the social/relationship domain (especially indicators relating to relationships with parents and other family members) and the health domain (especially the indicator for very high psychological distress). The coefficients for IPV come from fixed-effects logistic regression models. They show that the within-woman impacts of past-year IPV were especially pronounced in the social/relationship, health, and material domains. There was no evidence of within-woman impacts of past-year IPV on the employment or education domains.

A third set of additional analyses examined the unique impacts of different types of childhood DFSV (witnessing violence against a parent, sexual abuse, physical abuse, and psychological abuse) on IPV and MDP in young adulthood. The results of these analyses are displayed in Appendix 4. All forms of childhood DFSV were uniquely associated with significantly higher odds of experiencing moderate to high coercive control in young adulthood. Odds ratios were largest for childhood sexual abuse, which was associated with two times higher odds of moderate coercive control and 2.4 times higher odds of high coercive control compared to no IPV. Controlling for IPV and other covariates, the odds of being in MDP often during young adulthood were significantly higher among women who experienced sexual abuse, psychological abuse, and physical abuse growing up. The largest odds ratios were observed for childhood sexual and psychological abuse. Controlling for the other three forms of abuse, there was no significant association between witnessing violence against a parent during childhood and multidimensional poverty frequency in young adulthood.

## Discussion

In this study, we set out to generate robust and generalizable evidence on the longitudinal associations between violence and young women's unfreedom. Drawing on the work of development economists (Alkire & Foster, 2011; Nussbaum, 2005; Sen, 2001), we operationalized unfreedom using a measure of multidimensional poverty. While most prior studies on the impacts of gender-based violence have analyzed outcomes such as mental health and financial hardship separately, emerging evidence suggests that violence impacts women across multiple life domains simultaneously (see e.g., Gilroy et al., 2020; Sharp-Jeffs et al., 2018). These domains are experienced by women as closely intertwined (Ford-Gilboe et al., 2009), with the resulting “whole” arguably greater than the sum of its parts. Ultimately, accumulated deprivations reduce women's space for action (Stark, 2007), curtailing their freedom to realize their potential and live the life of their choosing. Crucially, without access to their full agentic power, it is difficult for women to take their place as equal citizens in political and public life (Nussbaum, 2005; Stark, 2007)—and thus to disrupt the reproduction of the gender order that oppresses them. As such, gender-based violence not only limits the freedom of individual survivors, it also plays a crucial role in reproducing the collective unfreedom of all women.

We found strong evidence that the damage caused by childhood exposure to DFSV reverberates through young women's lives. Compounding the disadvantage of women exposed to violence growing up was their elevated risk of revictimization at the hands of intimate partners in young adulthood. Exposure to violence of a greater breadth in childhood not only increased the risk of multidimensional poverty in adulthood, but also increased the risk of more severe IPV—which in turn further increased multidimensional poverty risk.

For example, after accounting for other covariates, the predicted probability of being in MDP at least once across the period of observation was approximately 11% for women who grew up free from violence and did not report any intimate partner violence, compared to 31% for women who grew up free from violence and reported high coercive control, and 64% for women who experienced three to four forms of DFSV growing up and reported high coercive control in young adulthood. For some women, the state of unfreedom was more than fleeting. Almost 5% of our sample were in MDP more often than not across the period of observation. The risk of falling into this category was significantly higher for women who experienced more severe violence in childhood and young adulthood. On average, the predicted probability of being in MDP often was 1.9% for women who grew up free from violence and did not report any intimate partner violence, compared to 12.7% for women who experienced two forms of DFSV growing up and reported moderate coercive control in young adulthood, and 24.6% for women who experienced three to four forms of DFSV growing up and reported high coercive control in young adulthood.

Results of a fixed-effects model provided additional, robust evidence that IPV directly increased women's risks of multidimensional poverty over and above the effects of childhood victimization and other time-invariant, individual characteristics. The odds that a woman would be in multidimensional poverty was more than one-and-a-half times higher when she had experienced moderate to high coercive control in the past year compared to years when she had not experienced any IPV. Additional analyses showed that the within-woman effects of intimate partner violence were most strongly felt in the social/relationship, health, and material domains. For example, women were 2.5 times as likely to report very high psychological distress, and twice as likely to find it always difficult/impossible to manage on their income and feel very/extremely stressed about their relationships with friends, in years when they reported high coercive control compared to years when they reported no IPV. These findings reinforce that the deleterious impacts of violence are experienced in more than one domain. Altogether, our findings provide strong quantitative support for Stark’s (2007) conceptualization of coercive control as a liberty crime—a crime against women's freedom.

### Limitations and Future Directions

Our measure of unfreedom suffered some data-driven limitations that should be kept in mind when interpreting our results. The only items consistently available in our dataset for the social/relationships domain of multidimensional poverty measured stress about relationships with family and friends. While relevant, these measures do not capture every important aspect of the social domain. Perceived social support—knowing there are people to turn to for emotional, practical, and financial help—is arguably an important material precondition for human freedom. Further, measures of unfreedom should not only include support available through interpersonal relationships, but from the community and state more broadly (see Tolmie et al., 2024). For example, a woman's degree of unfreedom will be heightened if she perceives that she will not receive adequate physical protection from the police, financial support from the state, and justice through the legal system upon leaving a violent relationship. The laws and policies that shape the availability of such supports (or lack thereof) are material processes of the gender order, and they clearly play a key role in reproducing women's unfreedom and facilitating men's ongoing perpetration of gender-based violence.

In addition to the above, a lack of appropriate data meant we were unable to capture the subjective elements of unfreedom—what Stark (2007) refers to as women's psychological entrapment. Feeling blocked or trapped, a lack of autonomy over one's day to day activities and decisions, and a perceived lack of control over one's life—all of these are potentially important inclusions. Further, there may be other crucial aspects of women's psychological entrapment that the multidimensional poverty framework has not identified. Arguably, the gender order not only distributes material resources inequitably between women and men, but also psychological resources such self-perceived entitlement and responsibility. Internalized sexism, shame, low self-esteem, and feelings of dependency: these are other socially produced, psychological aspects of women's unfreedom that future research might explore.

Future research should also build on our study by examining the outcomes of women at the intersection of different social identities. Intersectionality theory (e.g., Crenshaw, 1991) highlights racism, ableism, heterosexism, and colonization as powerful sources of oppression that intersect with sexism to shape women's experiences of both violence and poverty. As is the case in the United States (Rosay, 2016), First Nations women experience some of the highest rates of violence victimization in Australia. For example, while they make up approximately 3.3% of the nation's population, Indigenous Australians accounted for 28% of hospitalizations due to domestic and family violence between 2010 and 2019 (Australian Institute of Health and Welfare, 2021). The ALSWH dataset that we analyzed in this study does not contain detailed information on race, ethnicity, or First Nations status—a deliberate decision by the study's custodians to protect women's privacy and respect the wishes of First Nations communities. This is a limitation of our study that future research could address.

### Policy Implications

Our findings reiterate that ameliorating the harmful impacts of violence on women is a human rights imperative requiring a multifaceted approach. Supporting women to access the material resources they need to live safely and independently of their violent partners—including facilitating access to emergency funds, affordable housing, labor market opportunities, and childcare support—offers an important avenue for individual-level intervention (Kuskoff et al., 2022). Crucially, such supports must be designed and implemented in ways that account for the coinciding physical, psychological, and social harms that gender-based violence inflicts. Further, while such supports will be critical for enabling women to “flee” from their violent partners, they must extend beyond periods of crisis and into the medium- and long-term. This will give women the best chance to maintain their separation from the perpetrator and overcome the myriad forms of disadvantage that have hitherto dominated their lives.

### Conclusion

Our study has provided robust and generalizable evidence of a process of cumulative disadvantage, whereby early-life experiences of DFSV lead to the accumulation of further risks of violence and multidimensional poverty across young women's lives. We found that exposure to more severe DFSV during childhood increased the risks of deprivation across all five domains measured: material, employment, education, health and social/relationships. Not surprisingly, it also increased the risk of multidimensional poverty (i.e., deprivations across multiple domains simultaneously). In addition, exposure to more severe DFSV during childhood increased the risk of experiencing more severe intimate partner violence—characterized by high levels of coercive control—in young adulthood. In turn, these experiences of intimate partner violence further increased the risk of deprivation in the material, health and social/relationship domains, and the risk of multidimensional poverty overall.

A key takeaway of our study is that, for many women, the road to unfreedom begins in childhood. The policy implications of our findings are clear. First, there must be an increased investment in protecting children from all forms of DFSV. The costs of not doing so are severe and long-lasting. Second, victim-survivors of gender-based violence must have access to services that support their functionings in all life domains—physical and mental health, material wellbeing, employment, education, and social networks and relationships—with the end goal that their full agentic power be restored. Ultimately, freedom is a social product (Sen, 2001), and “people, especially women, are not free if they are left alone by a lazy state” (Nussbaum, 2005, p. 176). An equitable distribution of freedom will only be achieved when gender-based violence has been eliminated. The results of our study make it clear that, even in a relatively advantaged and gender-equal country such as Australia, this task is as urgent as ever.

## Supplemental Material

sj-docx-1-vaw-10.1177_10778012251347607 - Supplemental material for The Road to Unfreedom: Violence and Multidimensional Poverty Among Young Australian Women Supplemental material, sj-docx-1-vaw-10.1177_10778012251347607 for The Road to Unfreedom: Violence and Multidimensional Poverty Among Young Australian Women by Alice Campbell, Ella Kuskoff, Janeen Baxter and Deborah Loxton in Violence Against Women

## Notes

1. Frye (1983, p. 4) describes oppression as “the experience of being caged in: all avenues, in every direction are blocked or booby trapped” while Young (2021/2004: pp. 92–93) refers to all oppressed people as sharing “some inhibition of their ability to develop and exercise their capacities”. We view these understandings of oppression as synonymous with the concept of unfreedom as employed in our paper. A comprehensive theorisation of the relationship between unfreedom and oppression can be found in Drydyk (2021).

2. According to Sharp-Jeffs et al. (2018), the concept of “space for action” was developed by Kelly (2003) and inspired by the earlier work of Lundgren (1998). Broadly, it refers to the necessary preconditions (e.g., social, psychological and material resources) for women to be able to act freely according to their own needs and values, rather than in response to external constraints.

3. According to Loxton et al. (2019), 70% of the cohort were recruited via Facebook and an additional 4.9% via advertisements on other websites/social media including Twitter, Tumblr, and various blogs.

4. The Alkire-Foster method can be used to generate several policy-relevant metrics such as the headcount ratio, intensity of poverty, and the adjusted poverty index. Comprehensive descriptions of the method and all its applications are beyond the scope of this paper. Instead, we refer readers to Alkire and Foster (2011) and Alkire et al. (2015) for further information.

5. While it is standard for U.S. studies to collect data on race, this is not the case in Australia where cultural and linguistic diversity is more often the focus and is usually captured via country of birth. However, Australia is not unlike the United States in having serious problems with racism, which especially affects First Nations (Aboriginal and Torres Strait Islander) people. As we state in our discussion, investigating violence and multidimensional poverty in the lives of these women is an important avenue for future research.

6. Ideally, we would have included household's past year wealth status, and husband/partner's past year employment status as covariates, but these were not available in the dataset.

7. In contrast to fixed-effects models, random-effects models do allow for the estimation of coefficients for time-invariant variables. However, random-effects models are also prone to producing biased estimates if the assumption of no correlation between individual unobserved heterogeneity and the independent variables is violated. We estimated random-effects models but found evidence that this assumption was violated; therefore, we do not report the results here.

## References

[bibr1-10778012251347607] Alkire S. Foster A. (2011). Counting and multidimensional poverty measurement. Journal of Public Economics, 95(7-8), 476–487. 10.1016/j.jpubeco.2010.11.006

[bibr2-10778012251347607] Alkire S. Roche J. M. Ballon P. Foster J. Santos M. E. Seth S. (2015). Multidimensional poverty measurement and analysis. Oxford University Press.

[bibr3-10778012251347607] Alkire S. Santos M. E. (2013). A multidimensional approach: Poverty measurement & beyond. Social Indicators Research, 112(2), 239–257. 10.1007/s11205-013-0257-3

[bibr4-10778012251347607] Australian Bureau of Statistics. (2023). Childhood abuse. ABS. https://www.abs.gov.au/statistics/people/crime-and-justice/childhood-abuse/latest-release

[bibr5-10778012251347607] Australian Institute of Health and Welfare. (2021). Examination of hospital stays due to family and domestic violence 2010-11 to 2018-19. AIHW. https://www.aihw.gov.au/reports/family-domestic-and-sexual-violence/examination-of-hospital-stays-due-to-family-and-do/summary

[bibr6-10778012251347607] Brännström L. Vinnerljung B. Forsman H. Almquist Y. B. (2017). Children placed in out-of-home care as midlife adults: Are they still disadvantaged or have they caught up with their peers? Child Maltreatment, 22(3), 205–214. 10.1177/1077559517701855 28378598 PMC5497936

[bibr7-10778012251347607] Brownell P. (2024). Trauma theory and abuse, neglect and violence across the life course. Journal of Interpersonal Violence, 39(19-20), 4041–4064. 10.1177/08862605241264542 39254267

[bibr8-10778012251347607] Bunting L. Davidson G. McCartan C. Hanratty J. Bywaters P. Mason W. Steils N. (2018). The association between child maltreatment and adult poverty–A systematic review of longitudinal research. Child Abuse & Neglect, 77, 121–133. 10.1016/j.chiabu.2017.12.022 29346067

[bibr9-10778012251347607] Butler N. Quigg Z. Bellis M. A. (2020). Cycles of violence in England and Wales: The contribution of childhood abuse to risk of violence revictimization in adulthood. BMC Medicine, 18(325), 1–13. 10.1186/s12916-020-01788-3 33190642 PMC7667802

[bibr10-10778012251347607] Cameron A. Tedds L. M. (2021). Gender-based violence, economic security, and the potential of basic income: A discussion paper. MPRA. https://mpra.ub.uni-muenchen.de/107478/1/MPRA_paper_107478.pdf

[bibr11-10778012251347607] Campbell C. (2009). Distinguishing the power of agency from agentic power: A note on Weber and the “black box” of personal agency. Sociological Theory, 27(4), 407–418. 10.1111/j.1467-9558.2009.01355.x

[bibr12-10778012251347607] Campbell A. Perales F. Baxter J. (2020). Sexual minority women in longitudinal survey research: Is attrition a problem? Archives of Sexual Behavior, 49, 1443–1461. 10.1007/s10508-020-01669-z 32270401

[bibr13-10778012251347607] Cervantes M. V. Sherman J. (2021). Falling for the ones that were abusive: Cycles of violence in low-income women’s intimate relationships. Journal of Interpersonal Violence, 36(13-14), NP7567–NP7595. 10.1177/0886260519829771 . 30755063

[bibr14-10778012251347607] Chamberlain C. Johnson G. (2013). Pathways into adult homelessness. Journal of Sociology, 49(1), 60–77. 10.1177/1440783311422458 .

[bibr15-10778012251347607] Childress S. (2013). A meta-summary of qualitative findings on the lived experience among culturally diverse domestic violence survivors. Issues in Mental Health Nursing, 34(9), 693–705. 10.3109/01612840.2013.791735 24004364

[bibr16-10778012251347607] Cicchetti D. (2013). Annual research review: Resilient functioning in maltreated children–past, present, and future perspectives. Journal of Child Psychology and Psychiatry, 54(4), 402–422. 10.1111/j.1469-7610.2012.02608.x 22928717 PMC3514621

[bibr17-10778012251347607] Crenshaw K. (1991). Mapping the margins: Intersectionality, identity politics, and violence against women of color. Stanford Law Review, 43(6), 1241–1299. 10.2307/1229039

[bibr18-10778012251347607] Dannefer D. (2003). Cumulative advantage/disadvantage and the life course: Cross-fertilizing age and social science theory. The Journals of Gerontology Series B: Psychological Sciences and Social Sciences, 58(6), 327–337. 10.1093/geronb/58.6.S327 14614120

[bibr19-10778012251347607] Drydyk J. (2021). Capability and oppression. Journal of Human Development and Capabilities, 22(4), 527–550. 10.1080/19452829.2021.1982880

[bibr20-10778012251347607] Elder G. H. Jr. (1995). The life course paradigm: Social change and individual development. In Moen P. Elder G. H. Jr. Lüscher K. (Eds.), Examining lives in context: Perspectives on the ecology of human development (pp. 211–262). American Psychological Association. 10.1037/10176-000

[bibr21-10778012251347607] Farber N. Miller-Cribbs J. E. (2014). Violence in the lives of rural, southern, and poor white women. Violence Against Women, 20(5), 517–538. 10.1177/1077801214535104 24920456

[bibr22-10778012251347607] Felitti V. J. Anda R. F. Nordenberg D. Williamson D. F. Spitz A. M. Edwards V. Marks J. S. (1998). Relationship of childhood abuse and household dysfunction to many of the leading causes of death in adults: The adverse childhood experiences (ACE) study. American Journal of Preventive Medicine, 14(4), 245–258. 10.1016/s0749-3797(98)00017-8 9635069

[bibr23-10778012251347607] Ford-Gilboe M. Wuest J. Varcoe C. Davies L. Merritt-Gray M. Campbell J. Wilk P. (2009). Modelling the effects of intimate partner violence and access to resources on women's health in the early years after leaving an abusive partner. Social Science & Medicine, 68(6), 1021–1029. 10.1016/j.socscimed.2009.01.003 19188012

[bibr24-10778012251347607] Frye M. (1983). The politics of reality: Essays in feminist theory. Crossing Press.

[bibr25-10778012251347607] Gilroy H. McFarlane J. Fredland N. Cesario S. Nava A. Maddoux J. (2018). Using a model of economic solvency to understand the connection between economic factors and intimate partner violence. Journal of International Women's Studies, 19(6), 305–325.

[bibr26-10778012251347607] Gilroy H. Nava A. McFarlane J. (2020). Developing a theory of economic solvency for women who have experienced intimate partner violence. Violence Against Women, 26(9), 955–971. 10.1177/1077801219853366 31190625

[bibr27-10778012251347607] Giraldo-Rodríguez L. Mino-León D. Aragón-Grijalva S. O. Agudelo-Botero M. (2022). The revictimization of older Mexican women: Understanding the accumulation of multiple victimizations throughout a lifetime. BMC Geriatrics, 22(41), 1–12. 10.1186/s12877-021-02734-5 35012475 PMC8751368

[bibr28-10778012251347607] Goodman L. A. Smyth K. F. Borges A. M. Singer R. (2009). When crises collide: How intimate partner violence and poverty intersect to shape women’s mental health and coping. Trauma, Violence, & Abuse, 10(4), 306–329. 10.1177/1524838009339754 19776085

[bibr29-10778012251347607] Gustavson K. von Soest T. Karevold E. Røysamb E. (2012). Attrition and generalizability in longitudinal studies: Findings from a 15-year population-based study and a Monte Carlo simulation study. BMC public Health, 12(918), 1–11. 10.1186/1471-2458-12-918 23107281 PMC3503744

[bibr30-10778012251347607] Hegarty K. Sheehan M. Schonfeld C. (1999). A multidimensional definition of partner abuse: Development and preliminary validation of the composite abuse scale. Journal of Family Violence, 14(4), 399–415. 10.1023/A:1022834215681

[bibr31-10778012251347607] Heim C. Shugart M. W. Craighead E. Nemeroff C. B. (2010). Neurobiological and psychiatric consequences of child abuse and neglect. Developmental Psychobiology, 52(7), 671–690. 10.1002/dev.20494 20882586

[bibr32-10778012251347607] Herrenkohl T. I. Fedina L. Roberto K. A. Raquet K. L. Hu R. X. Rousson A. N. Mason A. (2022). Child maltreatment, youth violence, intimate partner violence, and elder mistreatment: A review and theoretical analysis of research on violence across the life course. Trauma, Violence, & Abuse, 23(1), 314–328. 10.1177/1524838020939119 PMC10202370 32723166

[bibr33-10778012251347607] Johnson M. P. Leone J. M. Xu Y. (2014). Intimate terrorism and situational couple violence in general surveys: Ex-spouses required. Violence Against Women, 20(2), 186–207. 10.1177/1077801214521324 24504325

[bibr34-10778012251347607] Kääriälä A. Hiilamo H. (2017). Children in out-of-home care as young adults: A systematic review of outcomes in the Nordic countries. Children and Youth Services Review, 79, 107–114. 10.1016/j.childyouth.2017.05.030

[bibr35-10778012251347607] Kelly L. (2003). The wrong debate: Reflections on why force is not the key issue with respect to trafficking in women for sexual exploitation. Feminist Review, 73(1), 139–144. 10.1057/palgrave.fr.9400086

[bibr37-10778012251347607] Kuskoff E. Parsell C. Plage S. Ablaza C. Perales F. (2022). Willing but unable: How resources help low-income mothers care for their children and minimise child protection interventions. British Journal of Social Work, 52(7), 3982–3998. 10.1093/bjsw/bcac027

[bibr38-10778012251347607] Lacey R. E. Howe L. D. Kelly-Irving M. Bartley M. Kelly Y. (2022). The clustering of adverse childhood experiences in the Avon Longitudinal Study of Parents and Children: Are gender and poverty important? Journal of Interpersonal Violence, 37(5-6), 2218–2241. 10.1177/0886260520935096 32639853 PMC8918866

[bibr39-10778012251347607] Loxton D. Harris M. L. Forder P. Powers J. Townsend N. Byles J. Mishra G. (2019). Factors influencing web-based survey response for a longitudinal cohort of young women born between 1989 and 1995. Journal of Medical Internet Research, 21(3), e11286. 10.2196/11286 PMC6452283 30907739

[bibr40-10778012251347607] Loxton D. Powers J. Fitzgerald D. Forder P. Anderson A. Taft A. Hegarty K. (2013). The Community Composite Abuse Scale: Reliability and validity of a measure of intimate partner violence in a community survey from the ALSWH. Journal of Women’s Health Issues Care, 2(4), 2–7. 10.4172/2325-9795.1000115

[bibr41-10778012251347607] Loxton D. Tooth L. Harris M. L. Forder P. M. Dobson A. Powers J. Brown W. Byles J. Mishra G. (2018). Cohort profile: The Australian Longitudinal Study on Women’s Health (ALSWH) 1989–95 cohort. International Journal of Epidemiology, 47(2), 391–392. 10.1093/ije/dyx133 29025118

[bibr42-10778012251347607] Lundgren E . (1998). The hand that strikes and comforts: Gender construction and the tension between body and symbol. In Dobash R. E. Dobash R. P. (Eds.), Rethinking violence against women (pp. 169–196). Sage.

[bibr43-10778012251347607] Martinez A. Perales F. (2017). The dynamics of multidimensional poverty in contemporary Australia. Social Indicators Research, 130, 479–496. 10.1007/s11205-015-1185-1

[bibr44-10778012251347607] McLachlan F. (2023). The rurality of intimate partner femicide: Examining risk factors in Queensland. Violence Against Women, 30(6–7), 1683–1707. 10.1177/10778012231158105 36815208 PMC10998431

[bibr45-10778012251347607] McNamara P. (2015). Young people at risk of lifelong poverty: Youth homelessness in Australia. In Fernandez E. Zeira A. Vecchiato T. Canali C. (Eds.), Theoretical and empirical insights into child and family poverty. Children’s well-being: Indicators and research (vol. 10, pp. 217–238). Springer. 10.1007/978-3-319-17506-5_14

[bibr46-10778012251347607] Nussbaum M. C. (2005). Women's bodies: Violence, security, capabilities. Journal of Human Development, 6(2), 167–183. 10.1080/14649880500120509

[bibr47-10778012251347607] O’Connor J. Nepomnyaschy L. (2020). Intimate partner violence and material hardship among urban mothers. Violence Against Women, 26(9), 935–954. 10.1177/1077801219854539 31304881

[bibr48-10778012251347607] Op den Kelder R. Van den Akker A. L. Geurts H. M. Lindauer R. J. L. Overbeek G. (2018). Executive functions in trauma-exposed youth: A meta-analysis. European Journal of Psychotraumatology, 9(1), 1–25. 10.1080/20008198.2018.1450595 PMC7803075 33488998

[bibr49-10778012251347607] Perlman S. Fantuzzo J. (2013). Predicting risk of placement: A population-based study of out-of-home placement, child maltreatment, and emergency housing. Journal of the Society for Social Work and Research, 4(2), 99–113. 10.5243/jsswr.2013.7

[bibr50-10778012251347607] Pezzoli P. Pingault J. B. Eley T. C. McCrory E. Viding E. (2024). Causal and common risk pathways linking childhood maltreatment to later intimate partner violence victimization. Molecular Psychiatry, 30, 2027–2037. 10.1038/s41380-024-02813-0 39488656 PMC12015119

[bibr51-10778012251347607] Rosay A. B. (2016). Violence against American Indian and Alaska Native women and men: 2010 findings from the National intimate partner and sexual violence survey. National Institute of Justice. https://nij.ojp.gov/library/publications/violence-against-american-indian-and-alaska-native-women-and-men-2010-findings

[bibr52-10778012251347607] Rothenbühler M. Voorpostel M. (2016). Attrition in the Swiss Household Panel: Are vulnerable groups more affected than others? In Oris M. Roberts C. Joye D. Ernst Stähli M. (Eds.), Surveying human vulnerabilities across the life course (pp. 223–244). Springer.

[bibr53-10778012251347607] Saiepour N. Najman J. M. Ware R. Baker P. Clavarino A. M. Williams G. M. (2019). Does attrition affect estimates of association: A longitudinal study. Journal of Psychiatric Research, 110, 127–142. 10.1016/j.jpsychires.2018.12.022 30639918

[bibr54-10778012251347607] Sariaslan A. Kääriälä A. Pitkänen J. , et al. (2022). Long-term health and social outcomes in children and adolescents placed in out-of-home care. JAMA Pediatrics, 176(1), e214324. 10.1001/jamapediatrics.2021.4324 PMC8546624 34694331

[bibr55-10778012251347607] Scutella R. Wilkins R. Kostenko W. (2013). Intensity and persistence of individuals’ social exclusion in Australia. Australian Journal of Social Issues, 48(3), 273–298. 10.1002/j.1839-4655.2013.tb00283.x

[bibr56-10778012251347607] Sen A. (2001). Development as freedom. Oxford University Press.

[bibr57-10778012251347607] Sharp-Jeffs N. Kelly L. Klein R. (2018). Long journeys toward freedom: The relationship between coercive control and space for action—Measurement and emerging evidence. Violence Against Women, 24(2), 163–185. 10.1177/1077801216686199 29332542

[bibr58-10778012251347607] Stark E. (2007). Coercive control: The entrapment of women in personal life. Oxford University Press.

[bibr59-10778012251347607] Strand S. J. M. Storey J. E. (2018). Intimate partner violence in urban, rural, and remote areas: An investigation of offense severity and risk factors. Violence Against Women, 25(2), 188–207. 10.1177/1077801218766611 29623774

[bibr60-10778012251347607] Tolmie J. Smith R. Wilson D. (2024). Understanding intimate partner violence: Why coercive control requires a social and systemic entrapment framework. Violence Against Women, 30(1), 54–74. 10.1177/10778012231205585 37807727 PMC10666472

[bibr61-10778012251347607] van der Velden P. G. Das M. Contino C. van der Knaap L. M. (2021). From health to financial problems: Multiproblems among victims of partner and non-partner physical violence, and matched nonvictims. Journal of Interpersonal Violence, 36(21–22), 10527–10545. 10.1177/0886260519885915 31686594 PMC8581717

[bibr62-10778012251347607] Watson N. Wooden M. (2009). Identifying factors affecting longitudinal survey response. In Lynn P. (Ed.), Methodology of longitudinal surveys (pp. 157–182). John Wiley & Sons, Incorporated. 10.1002/9780470743874

[bibr63-10778012251347607] Wooldridge J. M. (2010). Econometric analysis of cross section and panel data. MIT press.

[bibr64-10778012251347607] Young I. M. (2021). Five faces of oppression. In Ferguson M. Valls A. (Eds.), Iris Marion young (pp. 92–111). Routledge. (Original work published in 2004).

