# Supplementary material for: The Road to Unfreedom: Violence and Multidimensional Poverty Among Young Australian Women
Source: Violence Against Women. 2025 Jun 11;32(8):2222–52. doi: 10.1177/10778012251347607 (PMC13083815; doi:10.1177/10778012251347607)
Supplement: sj-docx-1-vaw-10.1177_10778012251347607 - Supplemental material for The Road to Unfreedom: Violence and Multidimensional Poverty Among Young Australian Women [file sj-docx-1-vaw-10.1177_10778012251347607.docx]

**Online Appendices**

**Appendix 1**

**Table A1.** Variables used to construct multidimensional poverty index

| **Domain** | **Variable** | **Deprivation cut-off** |
| --- | --- | --- |
| **1. Material resources** | Has a low-income healthcare card | 1 = Yes  0 = No |
|  | Ability to manage on income:  “How do you manage on the income you have available?” | 1 = Impossible/difficult all the time  0 = Difficult some of the time/not too bad/easy |
|  | Financial stress:  “Over the last 12 months, how stressed have you felt about the following areas of your life? Money” | 1 = Extremely/very stressed  0 = Moderately/somewhat/not at all stressed |
| **2. Employment** | Unemployed >6 months | 1 = Yes  0 = No |
|  | Usual hours of work:  “In a usual week, how many hours do you spend doing paid work?” | 1 = 0 hours  0 = 1+ hours |
| **3. Education** | Highest educational qualification | 1 = Year 12 (high school) or less and not currently studying  0 = Tertiary qualification (attained or currently studying for) |
| **4. Health** | General health:  “In general, would you say your health is:” | 1 = Poor  0 = Fair/good/very good/extremely good |
|  | Mental health:  Total score on 10-item Kessler Psychological Distress Scale (K10: range = 10-50) | 1 = 30+ (very high psychological distress)  0 = 10-29 |
| **5. Social/**  **Relationships** | Relationship with parents:  “Over the last 12 months, how stressed have you felt about the following areas of your life? Relationship with parents | 1 = Extremely/very stressed  0 = Moderately/somewhat/not at all stressed |
|  | Relationship with family:  “Over the last 12 months, how stressed have you felt about the following areas of your life? Relationship with other family members | 1 = Extremely/very stressed  0 = Moderately/somewhat/not at all stressed |
|  | Relationship with friends:  “Over the last 12 months, how stressed have you felt about the following areas of your life? Relationship with friends.” | 1 = Extremely/very stressed  0 = Moderately/somewhat/not at all stressed |

**Appendix 2: Data-driven weighting schemes for MDP index**

**Table A2**. Factor loadings and weights for multidimensional poverty index domains and indicators under two alternative specifications

|  | Alternative index 1 | |
| --- | --- | --- |
| Domain | Factor loading | Weight |
| Material | .731 | .255 |
| Employment | .558 | .194 |
| Education | .301 | .105 |
| Health | .696 | .243 |
| Social/relationships | .582 | .203 |
|  | Alternative index 2 | |
| Indicator | Factor loading | Weight |
| Always difficult/impossible to manage on income | .6838 | .1109 |
| Very/extremely stressed about money | .6710 | .1089 |
| Has a low-income healthcare card | .4911 | .0797 |
| Has been unemployed for more than 6 months | .6000 | .0973 |
| Works 0 hours in a usual week | .6161 | .1000 |
| Highest educational qualification is Year 12 or less | .2737 | .0444 |
| Poor self-rated health | .5468 | .0887 |
| Very high psychological distress | .6846 | .1111 |
| Very/extremely stressed about relationship with parents | .5131 | .0832 |
| Very/extremely stressed about relationship with other family | .5414 | .0878 |
| Very/extremely stressed about relationship with friends | .5421 | .0880 |

**Table A2.1**. Coefficients from fixed-effects logistic regression models of two specifications of multidimensional poverty index compared to original index

|  | Original index | Alternative index 1 | Alternative index 2 |
| --- | --- | --- | --- |
|  | *OR (SE)* | *OR (SE)* | *OR (SE)* |
| Past-year IPV (ref = none) |  |  |  |
| IPV with no/low coercive control | 1.103 (.090) | 1.138 (.091) | 1.236^**^ (.091) |
| Moderate coercive control | 1.698^***^ (.177) | 1.612^***^ (.170) | 1.673^***^ (.166) |
| High coercive control | 1.581^**^ (.232) | 1.887^***^ (.273) | 1.987^***^ (.273) |
| Age | .500^***^ (.078) | .523^***^ (.082) | .542^***^ (.079) |
| Age-squared | 1.011^**^ (.003) | 1.010^**^ (.003) | 1.009^**^ (.003) |
| Location of residence (ref = major city) |  |  |  |
| Inner regional area | 1.338^**^ (.149) | 1.276^*^ (.141) | 1.256^*^ (.133) |
| Rural/remote area | 1.003 (.166) | 1.028 (.176) | .941 (.150) |
| Overseas | .876 (.251) | .710 (.210) | .920 (.232) |
| *N* Observations | 9, 417 | 9,390 | 10,927 |
| *N* Individuals | 1, 981 | 1,965 | 2,284 |

*Notes*. Australian Longitudinal Study on Women’s Health. Women born 1989-1995. Data from Waves 1-6 (2013, 2014, 2015, 2016, 2017, 2019). Original index = indicators and domains weighted equally. Alternative index 1 = domains weighted according to their factor loadings. Alternative index 2 = indicators weighted according to their factor loadings IPV = Intimate partner violence. Statistical significance: * *p* < .05, ** *p* < .01, ^***^ *p* < .001. Wald tests were used to compare coefficients from the original index to those from the two alternative indices. No significant differences were found.

**Appendix 3: Impact of violence on separate MDP indicators**

**Table A3.** Odds ratios for violence variables from cross-sectional logistic regression models (Wave 1) and fixed-effects logistic regression models (Waves 1-6) of multidimensional poverty indicators

|  | Independent variables | | | | | | |
| --- | --- | --- | --- | --- | --- | --- | --- |
|  | Childhood exposure to DFSV (reference = none)^a^ | | |  | Past-year IPV (reference = none)^b^ | | |
|  | 1 form | 2 forms | 3-4 forms |  | No/low CC | Moderate CC | High CC |
| Dependent variables | *OR (SE)* | *OR (SE)* | *OR (SE)* |  | *OR (SE)* | *OR (SE)* | *OR (SE)* |
| Always difficult/impossible to manage on income | 1.483^***^ (.088) | 1.420^***^ (.108) | 1.932^***^ (.158) |  | 1.181^**^ (.065) | 1.467^***^ (.116) | 1.933^***^ (.233) |
| Very/extremely stressed about money | 1.457^***^ (.076) | 1.687^***^ (.115) | 2.119^***^ (.168) |  | 1.315^***^ (.065) | 1.649^***^ (.123) | 1.839^***^ (.218) |
| Has a low-income healthcare card | 1.204^***^ (.066) | 1.428^***^ (.099) | 1.716^***^ (.134) |  | .886^*^ (.052) | 1.133 (.098) | 1.408^*^ (.197) |
| Has been unemployed for more than 6 months | 1.154 (.093) | 1.620^***^ (.152) | 1.746^***^ (.181) |  | .986 (.078) | 1.172 (.131) | 1.160 (.190) |
| Works 0 hours in a usual week | 1.264^***^ (.079) | 1.612^***^ (.124) | 1.812^***^ (.155) |  | .916 (.060) | .824 (.082) | .988 (.145) |
| Highest educational qualification is Year 12 or less | 1.490^***^ (.105) | 1.696^***^ (.148) | 1.963^***^ (.185) |  | 1.121 (.095) | 1.111 (.123) | 1.271 (.222) |
| Poor self-rated health | 1.446^*^ (.231) | 2.454^***^ (.413) | 2.174^***^ (.412) |  | 1.105 (.135) | 1.265 (.199) | 1.003 (.245) |
| Very high psychological distress | 1.824^***^ (.115) | 2.098^***^ (.164) | 3.161^***^ (.265) |  | 1.533^***^ (.091) | 2.016^***^ (.170) | 2.566^***^ (.330) |
| Very/extremely stressed about relationship with parents | 2.172^***^ (.162) | 3.154^***^ (.272) | 3.798^***^ (.357) |  | 1.043 (.072) | 1.277^*^ (.122) | 1.393^*^ (.193) |
| Very/extremely stressed about relationship with other family | 1.936^***^ (.192) | 2.291^***^ (.267) | 3.308^***^ (.389) |  | 1.041 (.090) | 1.519^***^ (.176) | 1.578^**^ (.245) |
| Very/extremely stressed about relationship with friends | 1.352^***^ (.119) | 1.393^**^ (.154) | 2.006^***^ (.223) |  | 1.356^***^ (.104) | 1.455^***^ (.154) | 2.046^***^ (.308) |

*Notes*. Australian Longitudinal Study on Women’s Health. Women born 1989-1995. ^a^ Impacts of childhood exposure to DFSV on each indicator estimated using cross-sectional logistic regression models (Wave 1). Covariates included in models = past-year IPV, childhood poverty, age, age-squared, country of birth, and location of residence. ^b^ Impacts of past-year IPV on each indicator estimated with fixed-effects logistic regression models (Waves 1-6). Covariates included in models = age, age-squared, and location of residence. IPV = intimate partner violence. DFSV = domestic, family, and sexual violence. CC = coercive control. Statistical significance: * *p* < .05, ** *p* < .01, *** *p* < .001

**Appendix 4: Impact of different forms of childhood DFSV on IPV severity and MDP frequency**

**Table A4.** Relative risk ratios from multinomial logit model of intimate partner violence severity as a function of different forms of childhood violence

|  | Most severe IPV reported across all study waves  (reference = none reported) | | | | | |
| --- | --- | --- | --- | --- | --- | --- |
|  | IPV with no/low coercive control | | Moderate coercive control | | High coercive control | |
|  | *RRR* | *95%CI* | *RRR* | *95%CI* | *RRR* | *95%CI* |
| Witnessed violence against a parent/s | 1.159 | .99, 1.36 | 1.268^*^ | 1.04, 1.54 | 1.837^***^ | 1.44, 2.34 |
| Sexually abused | 1.394^***^ | 1.20, 1.61 | 2.026^***^ | 1.71, 2.41 | 2.436^***^ | 1.96, 3.03 |
| Psychologically abused | 1.443^***^ | 1.26, 1.65 | 1.606^***^ | 1.35, 1.91 | 1.523^***^ | 1.19, 1.94 |
| Physically abused | 1.263^*^ | 1.05, 1.52 | 1.612^***^ | 1.30, 2.00 | 2.120^***^ | 1.62, 2.78 |

*Notes*. Australian Longitudinal Study on Women’s Health. Women born 1989-1995. Data from Waves 1-6 (2013, 2014, 2015, 2016, 2017, 2019). IPV = Intimate partner violence. Covariates included in model (coefficients not shown) = age, country of birth, location of residence, and childhood poverty. Statistical significance: ^*^ *p* < .05, ^***^ *p* < .001

**Table A4.1.** Relative risk ratios from multinomial logit model of multidimensional poverty frequency as a function of different forms of childhood violence

|  | Multidimensional poverty frequency  (reference = never) | | | | | |
| --- | --- | --- | --- | --- | --- | --- |
|  | Rarely | | Sometimes | | Often | |
|  | *RRR* | *95%CI* | *RRR* | *95%CI* | *RRR* | *95%CI* |
| Witnessed violence against a parent/s | 1.221 | .99, 1.51 | 1.265^*^ | 1.02, 1.57 | 1.020 | .80, 1.30 |
| Sexually abused | 1.822^***^ | 1.51, 2.21 | 1.951^***^ | 1.61, 2.37 | 2.550^***^ | 2.06, 3.15 |
| Psychologically abused | 1.534^***^ | 1.27, 1.85 | 1.699^***^ | 1.39, 2.07 | 2.543^***^ | 2.02, 3.20 |
| Physically abused | 1.337^*^ | 1.06, 1.69 | 1.321^*^ | 1.04, 1.68 | 1.701^***^ | 1.32, 2.19 |

*Notes*. Australian Longitudinal Study on Women’s Health. Women born 1989-1995. Data from Waves 1-6 (2013, 2014, 2015, 2016, 2017, 2019). Covariates included in model (coefficients not shown) = age, country of birth, location of residence, and childhood poverty. Statistical significance: ^*^ *p* < .05, ^***^ *p* < .001
